# Assessment of D-Shaped Annulus of Mitral Valve in Patients with Severe MR Using Semi-Automated 4-Dimensional Analysis: Implications for Transcatheter Interventions

**DOI:** 10.3390/jcdd7040048

**Published:** 2020-11-01

**Authors:** N. Mai Vo, Suzanne E. van Wijngaarden, Nina Ajmone Marsan, Jeroen J. Bax, Victoria Delgado

**Affiliations:** Department of Cardiology, Heart Lung Center, Leiden University Medical Centre, Albinusdreef 2, 2300 RC Leiden, The Netherlands; n.m.vo@lumc.nl (N.M.V.); s.e.van_wijngaarden@lumc.nl (S.E.v.W.); n.ajmone@lumc.nl (N.A.M.); j.j.bax@lumc.nl (J.J.B.)

**Keywords:** mitral valve, transesophageal echocardiography, 3-dimensional, transcatheter

## Abstract

The development of transcatheter mitral valve replacement therapies requires accurate post-processing analysis tools to provide D-shaped mitral annulus dimensions from 3-dimensional (3D) data. The agreement between two semi-automated, software packages to process 3D transesophageal echocardiography (TEE) data for the measurement of the mitral valve annulus dimensions was evaluated. 3DTEE data of patients with moderate–severe mitral regurgitation (MR) were postprocessed with semi-automated, vendor-independent (VI) software and vendor-specific (VS) software. Both post-processing software provided key measurements for the selection of transcatheter valve prosthesis size: annulus area, annulus circumference and the septal-to-lateral distance of the annulus. The intertrigonal distance was provided only by the VS software. The inter- and intra-observer agreements were assessed with Bland–Altman analysis. Of 105 patients (63.8 ± 11 years, 66% male) with MR, 28 had secondary MR, 45 fibroelastic deficiency, and 32 Barlow’s disease. Using VS software, the dimensions for the overall population were 16.1 ± 4.6 cm^2^ for annulus area, for circumference 14.4 ± 1.9 cm, intertrigonal distance 3.4 ± 0.5 cm and septal-to-lateral distance 3.8 ± 0.6 cm. Similar dimensions were obtained using VI software: 15.7 ± 4.6 cm^2^ for annulus area, 14.5 ± 2.0 cm for circumference, and 4.1 ± 0.6 cm for septal-to-lateral distance. The inter- and intra-observer agreement for both software programs was excellent. In conclusion, current post-processing software programs for 3DTEE data of the mitral valve annulus provide good reproducibility of key measurements to select the transcatheter prosthesis size.

## 1. Introduction

The development and success of transcatheter mitral valve implantation requires accurate imaging modalities to characterize the mitral valve anatomy and geometry. Unlike the aortic valve, which is rigid and most often circular, the mitral valve has an asymmetric and more flexible annulus that challenges the design of the transcatheter mitral valve prostheses [1]. Moreover, the subvalvular apparatus needs to be carefully evaluated to prevent left ventricular outflow tract (LVOT) obstruction after mitral valve implantation [1]. 

In addition, the mitral annular dimensions and dynamics change according to the underlying mechanisms of mitral regurgitation (MR). In secondary MR, the mitral annulus is more rigid and has smaller dimensions than that of patients with primary MR [2]. Furthermore, within primary MR, the various phenotypes lead to significant variations in mitral valve geometry and dynamics: patients with Barlow’s disease (BD) showed larger mitral annular dimensions than patients with fibroelastic deficiency (FED) [3,4]. 

In transcatheter mitral valve implantation and direct annuloplasty techniques, accurate assessment of the mitral valve annulus is key to selecting the prosthesis or band size. Currently, computed tomography remains the mainstay to measure the mitral valve annulus and assess the location of neighboring structures that can be damaged during the procedure (i.e., the circumflex coronary artery). However, this imaging technique is not free of hazards and in patients with severe renal function impairment for example, the use of contrast may further impair the renal function and the use of intravenous saline for renal protection should be used with caution as patients with severe MR may present with pulmonary edema. Prior to the use of computed tomography, 3-dimensional (3D) transesophageal echocardiography (TEE) may serve as a gatekeeper to refer the patients that may be candidates for transcatheter mitral valve implantation and direct annuloplasty techniques for further evaluation with computed tomography. 

Several postprocessing software programs for 3DTEE data sets are available. It is unknown whether this software is interchangeable and can provide a standardized, semiautomated assessment of the mitral valve annulus. In addition, a few studies investigated the feasibility and reproducibility of mitral annulus analysis with 3DTEE data in a per MR-etiology basis. In this study we evaluated the agreement between two semi-automated postprocessing data software programs that measure the annulus of the mitral valve in a standardized approach and investigated the difference between secondary MR, BD and FED.

## 2. Materials and Methods 

Patients with severe MR referred for 3DTEE were included in the present study. Based on the morphology of the mitral valve and mechanism of MR, the patients were divided into 3 groups: secondary MR, BD and FED. Secondary MR was defined as MR caused by tethering and restrictive motion of structurally normal mitral leaflets due to annulus dilation and left ventricular remodeling [5]. FED was defined as prolapse of a single segment of the mitral leaflets characterized by thinned tissue. BD was defined as prolapse of the anterior and posterior leaflets with diffuse thickening and excessive tissue [5,6]. 

Demographics, cardiovascular risk factors, heart failure symptoms (according to New York Heart Association (NYHA) classification) and medication use were collected in the departmental cardiology information system (EPDVision; Leiden University Medical Center, Leiden, The Netherlands) and retrospectively analyzed. 

Clinically acquired 3DTEE data were postprocessed with two commercially available software programs (4D Auto MVQ, GE Healthcare, Horten, Norway and 4D MV Assessment, 2.2, TomTec Imaging systems, Munich, Germany) to create a model of the mitral valve and to derive measurements of the mitral valve annulus. The agreement between measurements obtained with these two software programs was assessed. The institutional ethical committee approved this evaluation and waived the need for patient written informed consent for retrospective analysis of clinically collected data (project identification code: CME10/024/SH, date of approval: 1 March 2020, institutional ethics committee: Leiden University Medical Center). 

Three-dimensional TEE data were acquired with a commercially available ultrasound system (E95; GE Vingmed Ultrasound AS, Horten, Norway) equipped with a 4D transesophageal matrix array probe (GE 6VT-D ultrasound transducer). A 3D full volume of the mitral valve was acquired adjusting the sector width and depth to optimize spatial and temporal resolution. To further optimize the temporal resolution, a multi-beat acquisition of the 3D full volume of the mitral valve was used during breath-holding whenever possible. 

**Figure 1:**
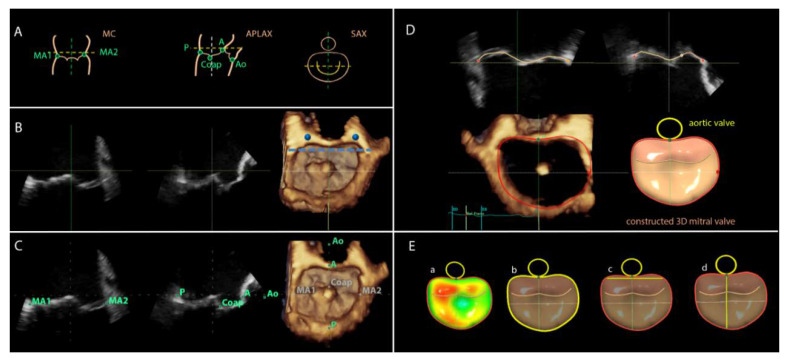
Overview of work flow of the 3D transesophageal echocardiographic semi-automated software of 4D Auto MVQ. (**A**) Graphical illustration of alignment and landmark placing. (**B**) Using the multiplanar reformation planes, the guidelines are aligned parallel to the 2D mitral annulus hinges in the mitral commissural view (MC) and the apical long axis view (APLAX). In the short axis view (SAX), the mitral annulus is shown in 3D. The trigones (marked in blue) are identified by moving the horizontal guideline until it intersects at the aorto–mitral border. (**C**) Landmarks are placed in MC and APLAX with direct 3D visualization in SAX for review: A, anterior annulus; Ao, aortic valve; Coap, leaflet coaptation; P, posterior annulus; MA1, anterolateral mitral annulus; MA2, posteromedial mitral annulus. (**D**) The software automatically defines the perimeter of the mitral annulus. The contours are shown for review and for manual adjustment if needed. (**E**) The software provides annulus dimensions of the D-shaped annulus, which is displayed in a reconstructed 3D model. Highlighted in multicolor: a, area. Highlighted in yellow: b, circumference (perimeter); c, intertrigonal distance; d, septal–lateral distance.

For image analyses the 3DTEE datasets were exported to an offline workstation (EchoPAC version 112.0.1; GE Vingmed Ultrasound AS) and were postprocessed with novel semi-automated software (4D Auto MVQ, GE Healthcare, Horten, Norway). From the 3D full volume dataset of the mitral valve, the mid-systolic frame was selected and the multiplanar reformation planes were aligned across the mitral commissures and perpendicular to obtain the mid-esophageal commissural (MC) and the apical longitudinal-axis (APLAX) views (Figure 1B). A third plane orthogonal to the MC and APLAX planes was oriented parallel to the lateral and medial trigones (Figure 1B). Subsequently, the landmarks were placed on the anterior, posterior, anterolateral and posteromedial points of the mitral annulus, the coaptation leaflet point and the aortic valve (Figure 1C). The software semi-automatically defined the perimeter of the mitral annulus and the area of the leaflets in the mid-systolic frame. The contours can be manually adjusted if needed. Thereafter, the software tracked the landmarks in all frames along the cardiac cycle to create a dynamic 3D mitral model. The contour tracking can be manually adjusted if needed on the reconstructed orthogonal planes (Figure 1D). After final approval of the reconstructed model, the software provided automated measurements of the D-shaped mitral annulus at mid-systole: annulus area (cm^2^) and perimeter (circumference, cm), intertrigonal distance (TT, cm), and septal-to-lateral (SL, cm) distance (Figure 1E). 

Similarly, the 3DTEE data were postprocessed with the 4D MV Assessment software (2.2, TomTec Imaging systems, Munich, Germany). After defining a mid-systolic frame on the 3D full volume dataset of the mitral valve, 3 parallel planes of the mid-esophageal APLAX view were provided. The plane where the anterior and posterior points of the mitral valve annulus, coaptation point and aortic valve can be placed was selected and the orthogonal plane showing the anterolateral and posteromedial commissures was displayed to set those landmarks. The software created a static model of the mitral valve annulus and leaflets by tracing the contours that can be manually corrected if needed. Subsequently, the contours tracked the movement of the mitral valve annulus and leaflets along the cardiac cycle creating a dynamic model of the mitral valve. The software permits overriding of the tracking with manual adjustments when needed. After approval of the model, the software provided several automated measurements of the D-shaped mitral annulus at mid-systole: annulus area (cm^2^), perimeter (circumference, cm) and SL distance (cm) [3]. The automated measurements of the mitral valve annulus obtained with the 4D Auto MVQ software were compared with the measurements obtained with the 4D MV Assessment software. 

Categorical variables are presented as numbers and percentages. Continuous variables with a normal distribution are presented as the mean ± standard deviation. Comparisons of categorical variables were evaluated using the χ2 test, whereas continuous data were compared using the one-way analysis of variance. Post-hoc analysis of continuous variables was performed with the Bonferroni test. Automated measurements of the mitral valve annulus provided by the 4D Auto MVQ and the 4D MV Assessment software were compared within each MR group (except for the intertrigonal distance which is only provided by the 4D Auto MVQ software). The limits of agreement and the mean difference between the 2 post-processing software programs were assessed with Bland–Altman analysis. The intra- and inter-observer agreement were assessed according to the intraclass correlation coefficient. The 95% confidence intervals were provided. A *p*-value <0.05 was considered statistically significant. Analyses were performed using SPSS software (Version 23.0. IBM Corp., Armonk, NY, USA). 

## 3. Results

Of 105 patients with severe MR (mean age of 63.8 ± 11 years, 66% male), 28 had secondary MR, 45 FED and 32 BD (Table 1). Patients with secondary MR were significantly older and had more frequently prior myocardial infarction, hypercholesterolemia, and diabetes mellitus. Most of the patients had NYHA II–IV heart failure symptoms. However, the group of patients with FED had more frequently NYHA I heart failure symptoms. The use of angiotensin converting enzyme inhibitors, angiotensin receptor blockers, beta-blockers, diuretics and statins were significantly higher among patients in the secondary MR compared with the FED and BD groups (Table 1).

The 3DTEE data of the D-shaped mitral valve annulus for all groups are shown in Table 2. Using the 4D Auto MVQ VS software, the dimensions for the overall population were: 16.1 ± 4.6 cm^2^ for the annulus area, 14.4 ± 1.9 cm for the perimeter, 3.4 ± 0.5 cm for the TT distance and 3.8 ± 0.6 cm for the SL distance. The dimensions obtained with the 4D MV Assessment were comparable and there were no statistically significant differences: 15.7 ± 4.6 cm^2^ for the annulus area, 14.5 ± 2.0 cm for the perimeter and 4.1 ± 0.6 cm for the SL distance (Table 2). Across the groups, the mitral annular dimensions of BD patients were significantly larger compared to secondary MR or FED (Table 2).

**Figure 2:**
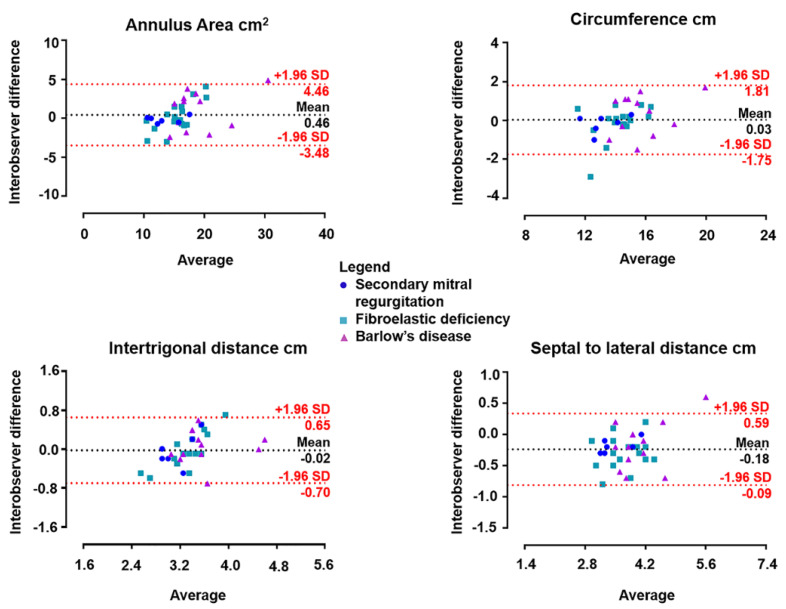
Bland–Altman plots of inter-observer difference using 4D Auto MVQ. Comparison of D-shaped annulus dimensions of mitral valve regurgitation with secondary MR, fibroelastic deficiency and Barlow’s disease.

The intra- and inter-observer agreement with 4D Auto MVQ was assessed in 36 randomly selected patients (7 secondary MR, 17 FED and 12 BD). Table 3 shows excellent intraclass correlation coefficients for the annulus measurements. Furthermore, the inter-observer reproducibility is shown with Bland–Altman plots for each mitral annulus dimension in Figure 2. The Bland–Altman plots illustrate the distribution of the mitral annulus assessments of secondary MR, BD and FED. For each MR mechanism, 4D Auto MVQ yielded similar measurements to those provided by the 4D MV Assessment software in all dimensions of the mitral valve annulus. 

## 4. Discussion

The present study showed a good agreement for the assessment of D-shaped mitral annulus between the novel 4D Auto MVQ VS (GE Healthcare) and the established 4D MV Assessment software (TomTec Imaging systems) in patients with severe MR. The intra- and inter-observer reproducibility using 4D Auto MVQ were excellent. Across the different MR mechanisms, the inter-observer reproducibility was excellent using 4D Auto MVQ. 

With the advent of transcatheter mitral valve interventions, automated measurements of the mitral valve annulus are necessary. The mitral annulus has shown to have a dynamic saddle shape that challenges the measurement of the various dimensions that are relevant for transcatheter valve ring and prosthesis size [1]. 

Computed tomography has been the imaging technique of first choice to assess the mitral valve annulus dimensions for patients who are candidates for transcatheter mitral valve implantation or repair (using mitral annuloplasty devices). Compared to TEE, computed tomography provides accurate information on the spatial relationship of the mitral annulus with neighboring structures that need to be considered in the selection of patients for transcatheter mitral valve interventions (i.e., location of the coronary sinus and circumflex coronary artery, calcification of the mitral annulus, LV outflow tract dimensions). However, many of the patients who are candidates for those therapies have associated comorbidities such as renal dysfunction that limit the widespread use of computed tomography with iodinated contrast. In addition, echocardiography is the imaging technique of first choice to determine the severity of MR and the underlying mechanism. 

Thus, performing the 3DTEE as a first imaging technique and being able to assess the mitral valve dimensions would help to decide which patients need further evaluation with computed tomography. In an effort to provide consistent measurements and reduce the impact of the inter-observer variability, several post-processing semi-automated software programs of 3DTEE datasets have been released. However, the consistency across the software programs is unclear. In addition, the 4D Auto MVQ software of GE Healthcare does report the D-shape of the mitral annulus and provides the intertrigonal distance, whereas 4D MV Assessment 2.2 software of TomTec Imaging systems does not provide that measurement but reports the entire perimeter of the mitral annulus. Although previous studies included heterogeneous populations with various MR mechanisms, none of the studies compared the agreement between software programs for evaluating the different mechanisms underlying MR [2,7]. 

The characteristics of the mitral annulus vary depending on the underlying mechanism of MR. This also accounts for the D-shaped annulus; various studies have confirmed that the D-shaped mitral annular dimensions in primary MR are larger than those of secondary MR [2,8]. 

In primary MR, two different phenotypes can be identified: FED, with a single scallop prolapsing or with a flail leaflet while the remaining scallops are often normal, versus BD with excessive and redundant valve tissue with myxomatous degeneration causing prolapse of multiple or all scallops. 3DTEE has demonstrated the different dynamics of the mitral valve annulus in these two entities. The mitral annulus dimensions of the patients with BD are larger compared to patients with FED and the dynamics of the mitral valve annulus were abnormal in patients with BD, leading to an increase in the mitral annulus area and intercommissural diameter in systole, while in patients with FED the dimensions of the mitral annulus remain relatively normal [4]. Characterization of the mitral annulus dynamics can have implications for transcatheter therapies and can be assessed with 3DTEE. In the present study, both 3DTEE postprocessing software programs demonstrated that patients with BD had larger mitral annulus dimensions than patients with FED and the agreement between both software programs was good. 

In secondary MR, the mitral annulus is dilated but is less dynamic during the cardiac cycle compared to primary MR. Similar to previous studies [3,4], the present study showed that the mitral annulus dimensions in patients with secondary MR are smaller compared to patients with BD causing primary MR and both 3DTEE postprocessing software programs provided comparable results. Therefore, the currently available 3DTEE postprocessing software programs could be used interchangeably to assess the mitral annulus dimensions. 

## 5. Conclusions

Mitral annulus dimensions assessment with 3DTEE datasets are reproducible and interchangeable between two semi-automated postprocessing software programs. In addition, the underlying etiology of MR does not impact on the accuracy of the measurements. 

## Figures and Tables

**Table 1:** Clinical characteristics.

Characteristics	Total N = 105	Secondary MR N = 28	FED N = 45	BD N = 32	*p*-Value
**Age, years**	63.8 ± 11.0	69.4 ± 9.3 ^§^	63.2 ± 10.3	59.7 ± 11.7 *	0.002
**Male, *n* (%)**	66 (62.9)	18 (64.3)	29 (64.4)	19 (59.4)	0.890
**BSA, m^2^**	2.0 ± 0.2	1.9 ± 0.18	2.0 ± 0.23	2.0 ± 0.19	0.283
**Prior MI, *n* (%)**	12 (11.4)	10 (35.7) ^†§^	1 (2.2)	1 (3.1)	<0.001
**Hypertension, *n* (%)**	42 (40.0)	12 (42.9)	18 (40.0)	12 (37.5)	0.917
**Hypercholesterolemia, *n* (%)**	21 (20.0)	12 (42.9) ^†§^	7 (15.6)	2 (6.3)	0.001
**Diabetes mellitus, *n* (%)**	14 (13.3)	9 (32.1) ^†§^	4 (8.9)	1 (3.1)	0.002
**Smoker, *n* (%)**	43 (41.0)	28 (71.4)	18 (40.0)	8 (25.0)	0.354
**NYHA class, *n* (%)**					
**I**	24 (22.9)	3 (10.7) ^§^	16 (35.6) *	5 (15.6)	0.024
**II**	43 (41.0)	12 (42.9)	14 (28.9)	17 (53.1)	0.152
**III/IV**	38 (36.2)	13 (46.4)	15 (33.3)	10 (31.3)	0.421
**ACEi/ARB, *n* (%)**	68 (64.8)	24 (85.7) ^§^	25 (55.6) *	19 (59.4)	0.023
**Beta-blocker, *n* (%)**	55 (52.4)	23 (82.1) ^†§^	18 (40)	14 (43.8)	0.001
**Ca-channel blocker, *n* (%)**	12 (11.4)	4 (14.3)	6 (13.3)	2 (6.3)	0.547
**Statin, *n* (%)**	41 (39)	21 (75.0) ^†§^	15 (33.3)	5 (15.6)	<0.001
**Diuretics, *n* (%)**	36 (34.3)	18 (64.3) ^†§^	12 (26.7)	6 (18.8)	<0.001

Mean ± SD and percentage reported. ACEi, angiotensin converting enzyme-inhibitor; ARB, angiotensin II receptor blocker; BD, Barlow’s disease; FED, fibroelastic deficiency; MI, myocardial infarction; MR, mitral regurgitation; NYHA class, New York Heart Association functional classification; * = *p*-value significant from FMR, ^§^ = *p*-value significant from BD, ^†^ = *p*-value significant from FED.

**Table 2:** Mitral annular dimensions.

Mitral Annular Dimensions	4D Auto MVQ					4D MV Assessment				
	Total N = 105	Secondary MR N = 28	FED N = 45	BD N = 32	*p*-Value	Total N = 105	Secondary MR N = 28	FED N = 45	BD N = 32	*p*-Value
**Area, cm^2^**	16.1 ± 4.6 (7.1–35.4)	13.9 ± 3.6 (7.1–25.5)	15.2 ± 3.2 (9.1–23.4)	19.6 ± 5.2 *^†^ (13.1–35.4)	**<0.001**	15.7 ± 4.6 (7.4–38.7)	13.9 ± 4.0 (7.4–27.5)	14.9 ± 2.7 (9.8–23.4)	18.4 ± 5.9 *^†^ (11.8–38.7)	**<0.001**
**Circumference, cm**	14.4 ± 1.9 (9.9–21.5)	13.4 ± 1.7 (9.9–18.2)	13.9 ± 1.4 (10.9–17.6)	15.9 ± 2.0 *^†^ (13.1–21.5)	**<0.001**	14.5 ± 2.0 (10.1–22.9)	13.5 ± 1.8 (10.1–19.1)	14.2 ± 1.3 (11.5–17.6)	15.8 ± 2.3 *^†^ (12.8–22.9)	**<0.001**
**TT distance, cm**	3.4 ± 0.5 (2.3–4.7)	3.2 ± 0.4 (2.4–4.1)	3.3 ± 0.4 (2.3–4.3)	3.7 ± 0.5 *^†^ (2.7–4.7)	**<0.001**	-	-	-	-	**-**
**SL distance, cm**	3.8 ± 0.6 (2.1–5.9)	3.6 ± 0.6 (2.1–5.3)	3.6 ± 0.5 (2.8–4.8)	4.2 ± 0.6 *^†^ (3.3–5.9)	**<0.001**	4.1 ± 0.6 (2.6–6.2)	3.9 ± 0.7 (2.6–6.0)	4.0 ± 0.5 (3.0–5.0)	4.4 ± 0.7 *^†^ (3.6–6.2)	**0.003**

Mean ± SD and range reported. BD, Barlow’s disease; FED, fibroelastic deficiency; MR, mitral regurgitation; SL, septal–lateral; TT, intertrigonal; * = *p*-value significant from FMR; ^†^ = *p*-value significant from FED.

**Table 3:** Intra- and inter-observer variability of assessment of mitral annular dimensions with 4D Auto MVQ.

Variable	Intra-Observer Agreement, *n* = 36	Inter-Observer Agreement, *n* = 36
**Area, cm^2^**	0.931 (CI: 0.869–0.930; *p* < 0.0001)	0.964 (CI: 0.930–0.982; *p* < 0.0001)
**Circumference, cm**	0.918 (CI: 0.845–0.957; *p* < 0.0001)	0.957 (CI: 0.916–0.978; *p* < 0.0001)
**TT distance, cm**	0.733 (CI: 0.535–0.854; *p* < 0.0001)	0.846 (CI: 0.697–0.921; *p* < 0.0001)
**SL distance, cm**	0.821 (CI: 0.343–0.933; *p* < 0.0001)	0.901 (CI: 0.511–0.965; *p* < 0.0001)

SL, septal–lateral, TT, intertrigonal.

## References

[1] De Backer O., Piazza N., Banai S., Lutter G., Maisano F., Herrmann H.C., Franzen O.W., Sondergaard L. (2014). Percutaneous transcatheter mitral valve replacement: An overview of devices in preclinical and early clinical evaluation. Circ. Cardiovasc. Interv..

[2] Mak G.J., Blanke P., Ong K., Naoum C., Thompson C.R., Webb J.G., Moss R., Boone R., Ye J., Cheung A. (2016). Three-Dimensional Echocardiography Compared with Computed Tomography to Determine Mitral Annulus Size before Transcatheter Mitral Valve Implantation. Circ. Cardiovasc. Imaging.

[3] van Wijngaarden S.E., Kamperidis V., Regeer M.V., Palmen M., Schalij M.J., Klautz R.J., Bax J.J., Ajmone Marsan N., Delgado V. (2018). Three-dimensional assessment of mitral valve annulus dynamics and impact on quantification of MR. Eur. Heart J. Cardiovasc. Imaging.

[4] Clavel M.A., Mantovani F., Malouf J., Michelena H.I., Vatury O., Jain M.S., Mankad S.V., Suri R.M., Enriquez-Sarano M. (2015). Dynamic phenotypes of degenerative myxomatous mitral valve disease: Quantitative 3-dimensional echocardiographic study. Circ. Cardiovasc. Imaging.

[5] Vahanian A., Alfieri O., Andreotti F., Antunes M.J., Baron-Esquivias G., Baumgartner H., Borger M.A., Carrel T.P., De Bonis M., Evangelista A. (2012). Guidelines on the management of valvular heart disease (version 2012): The Joint Task Force on the Management of Valvular Heart Disease of the European Society of Cardiology (ESC) and the European Association for Cardio-Thoracic Surgery (EACTS). Eur. J. Cardiothorac. Surg..

[6] Anyanwu A.C., Adams D.H. (2007). Etiologic classification of degenerative mitral valve disease: Barlow’s disease and fibroelastic deficiency. Semin. Thorac. Cardiovasc. Surg..

[7] Shanks M., Delgado V., Ng A.C., van der Kley F., Schuijf J.D., Boersma E., van de Veire N.R., Nucifora G., Bertini M., de Roos A. (2010). Mitral valve morphology assessment: Three-dimensional transesophageal echocardiography versus computed tomography. Ann. Thorac. Surg..

[8] Naoum C., Leipsic J., Cheung A., Ye J., Bilbey N., Mak G., Berger A., Dvir D., Arepalli C., Grewal J. (2016). Mitral Annular Dimensions and Geometry in Patients with Functional MR and Mitral Valve Prolapse: Implications for Transcatheter Mitral Valve Implantation. JACC Cardiovasc. Imaging.

